# Evidence for additional *FREM1* heterogeneity in Manitoba oculotrichoanal syndrome

**Published:** 2012-05-30

**Authors:** Robertino Karlo Mateo, Royce Johnson, Ordan J. Lehmann

**Affiliations:** 1 Department of Medical Genetics, University of Alberta, Edmonton, Canada; 2 Department of Ophthalmology, University of Alberta, Edmonton, Canada

## Abstract

**Purpose:**

Manitoba Oculotrichoanal (MOTA) syndrome is an autosomal recessive disorder present in First Nations families that is characterized by ocular (cryptophthalmos), facial, and genital anomalies. At the commencement of this study, its genetic basis was undefined.

**Methods:**

Homozygosity analysis was employed to map the causative locus using DNA samples from four probands of Cree ancestry. After single nucleotide polymorphism (SNP) genotyping, data were analyzed and exported to PLINK to identify regions identical by descent (IBD) and common to the probands. Candidate genes within and adjacent to the IBD interval were sequenced to identify pathogenic variants, with analyses of potential deletions or duplications undertaken using the B-allele frequency and log_2_ ratio of SNP signal intensity.

**Results:**

Although no shared IBD region >1 Mb was evident on preliminary analysis, adjusting the criteria to permit the detection of smaller homozygous IBD regions revealed one 330 Kb segment on chromosome 9p22.3 present in all 4 probands. This interval comprising 152 SNPs, lies 16 Kb downstream of *FRAS1*-related extracellular matrix protein 1 (*FREM1*), and no copy number variations were detected either in the IBD region or *FREM1*. Subsequent sequencing of both genes in the IBD region, followed by *FREM1*, did not reveal any mutations.

**Conclusions:**

This study illustrates the utility of studying geographically isolated populations to identify genomic regions responsible for disease through analysis of small numbers of affected individuals. The location of the IBD region 16 kb from *FREM1* suggests the phenotype in these patients is attributable to a variant outside of *FREM1*, potentially in a regulatory element, whose identification may prove tractable to next generation sequencing. In the context of recent identification of *FREM1* coding mutations in a proportion of MOTA cases, characterization of such additional variants offers scope both to enhance understanding of *FREM1*’s role in cranio-facial biology and may facilitate genetic counselling in populations with high prevalences of MOTA to reduce the incidence of this disorder.

## Introduction

Manitoba Oculotrichoanal (MOTA) syndrome is a rare autosomal recessive disorder, first documented in the Island Lake region of Northern Manitoba [1]. Individuals of native Aboriginal descent (Canada’s First Nations peoples) exhibited ocular anomalies, most notably a fusion of the upper eyelid to the globe, known as subtotal cryptophthalmos or hidden eye. Associated phenotypes included facial anomalies with aberrant hair distribution extending below the brow, nasal dimpling, as well as ano-genital anomalies [2]. The existence of a similar disorder in the Inuit [3], who are ancestrally related to the First Nations, suggested a common genetic etiology. MOTA syndrome is phenotypically similar to Fraser Syndrome (FS), with common features including cryptophthalmos, nasal and genital anomalies [4]; however MOTA probands are less severely affected and to our knowledge do not exhibit cognitive impairment, syndactyly, renal, auricular, or limb defects.

Both disorders are autosomal recessively inherited [2]. Fraser syndrome cases are attributable to mutations in either *FRAS1* (Fraser syndrome 1) or *FREM2* (*FRAS1*-related extracellular matrix protein 2) [5-7], with these genes accounting for approximately 40% of cases. Other *FRAS/FREM* gene family members (*FREM1* and *FREM3*) form multi-protein complexes in the extracellular matrix that interact with *GRIP1*, (glutamate receptor-interacting protein 1) which serves to anchor *FRAS/FREM* proteins [8,9], and in which mutations were recently detected in FS probands [10]. Linkage analysis of Fraser Syndrome to the vicinity of *FREM1* (chromosome 9p22.3) was reported 5 years ago, however no disease causing mutations were identified [11]. More recently, homozygous *FREM1* mutations were shown in a Middle Eastern sibship [12] to be associated with a bifid nose, anorectal, and renal anomaly phenotype, but which lacked cryptophthalmos, suggesting that *FRAS*/*FREM* variants may contribute to a diverse spectrum of related disorders [13].

The *Fras/Frem*, and *Grip1* genes have been extensively studied in murine models, strains, collectively referred to as “bleb” mutants due to epidermal blistering during embryonic development [14-17]. These exhibit cryptophthalmos, syndactyly and renal defects that correspond with those phenotypes observed in FS patients. *Fras/Frem* genes, which are expressed in a tissue specific manner and encode proteins that are secreted into the extracellular matrix, regulate the bioavailability of growth factors during development [18] and so have key roles in tissue morphogenesis [19,20]. FRAS/FREM proteins contain chondroitin sulfate proteoglycan (CSPG) domains, and their tissue specific expression is thought to maintain epithelial-mesenchymal integrity during development via a mechanism similar to *CSPG4* (or *NG2*), directly binding collagens V and VI as well as fibroblast growth factor (*FGF*) and epidermal growth factor (*EGF*) [21,22].

At the commencement of this study MOTA syndrome was molecularly undefined, with no *FRAS/FREM* family members known to underlie MOTA. We used homozygosity mapping, an approach that permits mapping of genes responsible for autosomal recessive disorders [23-26]. Single nucleotide polymorphisms (SNPs) were used to identify regions that are Identical By Descent (IBD) in multiple affected individuals and so determine the genomic interval responsible for disease [27,28]. This methodology takes advantage of the geographically isolated nature of the First Nations community studied and MOTA’s reported inheritance pattern, enabling the molecular basis to be elucidated using a very small number of patient samples.

## Methods

### Patients and genomic DNA collection

**Figure 1:**
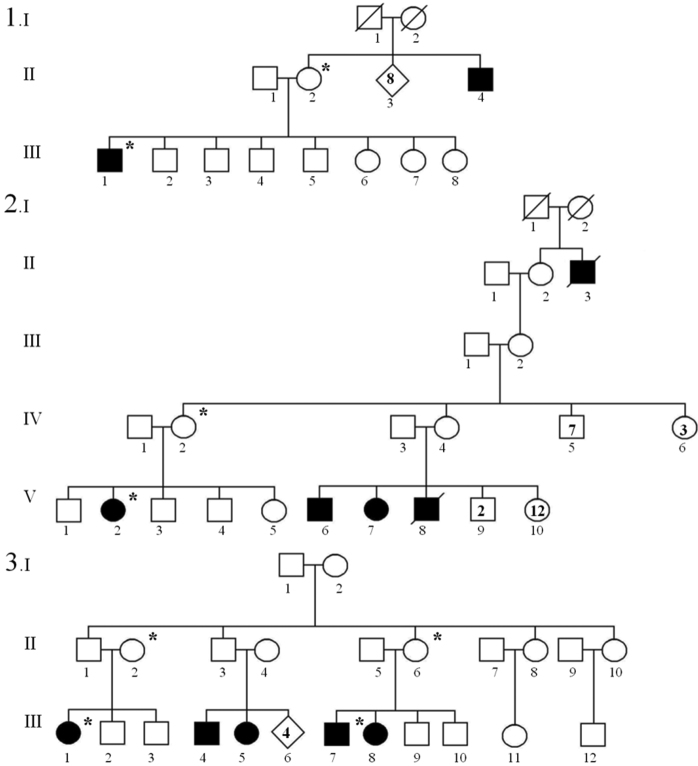
The three MOTA pedigrees exhibit an inheritance pattern compatible with autosomal recessive disease. Asterisks denote individuals that provided blood samples.

Affected individuals were derived from three pedigrees of Cree ancestry living in a geographically isolated region in Northern Alberta (Figure 1). Since the area is only accessible during the winter by ice roads, this was anticipated to result in high levels of consanguinity in the approximately 1,000 inhabitants. Blood samples were collected from four probands (1.III-1, 2.V-2, 3.III-1, and 3.III-7) and the unaffected parent (mother) that accompanied each child for oculoplastic surgery at the regional ophthalmic center, followed by genomic DNA extraction. Ethical approval was provided by the University of Alberta Hospital Health Research Ethics Board, and informed consent was obtained from all participants.

### Genotyping and homozygosity mapping

Genotyping was performed using a 610*-*Quad SNP array (Illumina Inc., San Diego, CA) comprising approximately 28,000 Copy Number Variant (CNV) probes and 592,000 single nucleotide polymorphisms (SNP*s)*, spa*c*ed at a mean distance of 1 SNP per 2.7 kb across the genome, and processed by deCODE geneti*c*s in Reykjavík, Iceland. Raw data were analyzed using GenomeStudio software (Illumina), non-Mendelian genotypes removed using the software’s Heritability Report algorithm, and then exported to PLINK v1.07 for homozygosity analysis [29]. Homozygous regions were then analyzed to define IBD intervals common among the probands. Initial homozygosity analysis performed using default PLINK parameters (homozygous region >1 Mb) did not identify an IBD interval common to all four probands. Subsequently, criteria were altered to permit detection of smaller homozygous segments (>300 kb) comprising at least 100 consecutive homozygous SNPs. In addition the percentage homozygosity of each genome was calculated using the total length of homozygous regions >300 kb divided by that of the autosomes (NCBI Build 36) [30].

### SNP visualization of genotype and CNV status

Two values were calculated from the array data to determine if any copy number variants were present. The first (B-Allele Frequency [BAF]) is derived from the relative ratio of fluorescent intensities of the two alleles at each SNP (Cy5 [green] A allele; Cy3 [red] B allele) with a heterozygous SNP having a BAF of 0.5, while homozygous SNPs are either 1 or 0. The second criterion used, is the logarithm of the ratio of the observed to the expected intensities at each SNP (Log_2_R ratio [LRR]), with deviations from zero (log_2_1) providing evidence of a CNV (deletion=-1, duplication=0.5, normal=0). Additional software (CnvPartition 3.1.6; Illumina) was used in parallel with LRR data to assign a CNV value for each SNP, and so detect any potential deletions or duplications.

### Candidate gene sequencing

The coding and splice junctions of three genes lying in or adjacent to the genomic region of interest (*FREM1*, cerberus 1 (*CER1*) [31], and zinc finger, DHHC-type containing 21 (*ZDHHC21*) [32]) were sequenced using published primers [12] or those designed with Primer3 (Appendix 1). Genomic DNA from a single affected individual (1.III-1) was used as template and sequence data generated (ABI Prism 3100, Applied Biosystems, Foster City, CA) was analyzed relative to the ENSEMBL reference sequence (Sequencher 4.6; GeneCodes, Madison, WI).

### Evolutionary conserved regions (ECRs) within the IBD region

In an effort to identify potential regulatory elements within the IBD interval, non-coding genomic sequences conserved in vertebrates were defined using ECR Browser [33], with appropriate correction for the different genomic builds (SNP array, Build 36, ECR Brower, Build 37) using the UCSC LiftOver tool [30]. Criteria consisted of ECRs with a minimum length of 90 bp and greater than 70% conservation of the human sequence against chimpanzee, rhesus monkey, cow, dog, opossum, rat, mouse, chicken, frog, pufferfish, or zebrafish genomes. ECRs conserved between human and *Xenopus* (Table 1) were selected for further analysis and sequenced with primers designed by Primer 3 (Appendix 2).

**Table 1:** Conserved regions identified within the 330 kb IBD region.

**ECR**	**Genomic position**	**Length (bp)**	**% identity**
1	14423929–14424022	94	89%
2	14443290–14443585	296	80%
3	14520772–14520883	112	71%
4	14521314–14521641	328	74%
5	14521719–14522534	816	78%
6	14522575–14522705	131	72%
7	14549013–14549211	199	71%

## Results

### Phenotypic analysis

**Figure 2:**
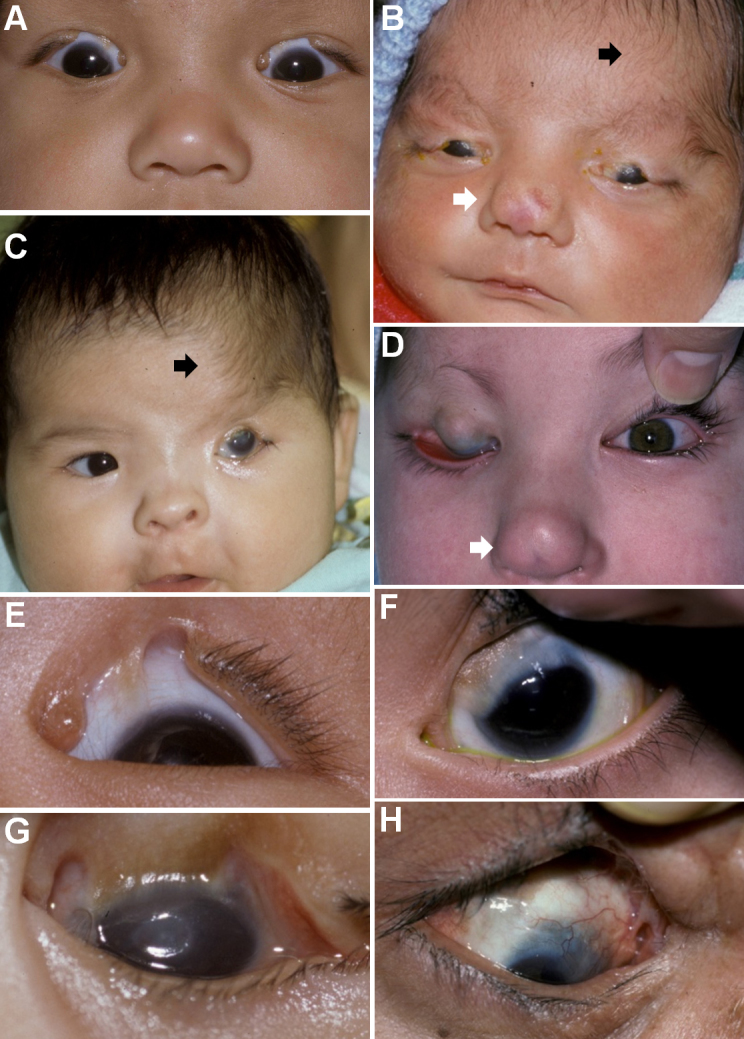
MOTA phenotypic spectrum in Albertan First Nations pedigrees. The oculo-facial phenotypes observed are diverse, ranging from isolated ocular anomalies to broader characteristics including dimpled noses (white arrows) and aberrant hair wedges where hair extends across the forehead to reach the eyebrow (black arrows). As evident from the montage, the ocular malformations can be bilateral (**A**, **B**) or unilateral (**C**, **D**), and vary in terms of the degree of lid involvement from isolated fusion (**D**) to abortive cryptophthalmos (**E**). Associated features include corneopalpebral synechiae (**E**, **F**), corneal opacification (**G**), and vascularization (**H**).

The four MOTA cases displayed a spectrum of ocular anomalies with considerable variation in phenotypic severity. There was a greater proportion of bilateral (n=3) than unilateral involvement (Figure 2), and cases with partial upper eyelid involvement most frequently affected the medial segment. Additional features included fusion of the eyelid to the cornea, which ranged in severity from total fusion (Figure 2D) to focal synechiae (Figure 2E,F), as well as frequent corneal opacification and corneal vascularisation (Figure 2G,H). Aberrant facial development was evident from extension of hair distribution from the scalp to reach the eyebrow (Figure 2B,C) as well as nasal dimpling (Figure 2B,D).

### Molecular analyses

**Figure 3:**
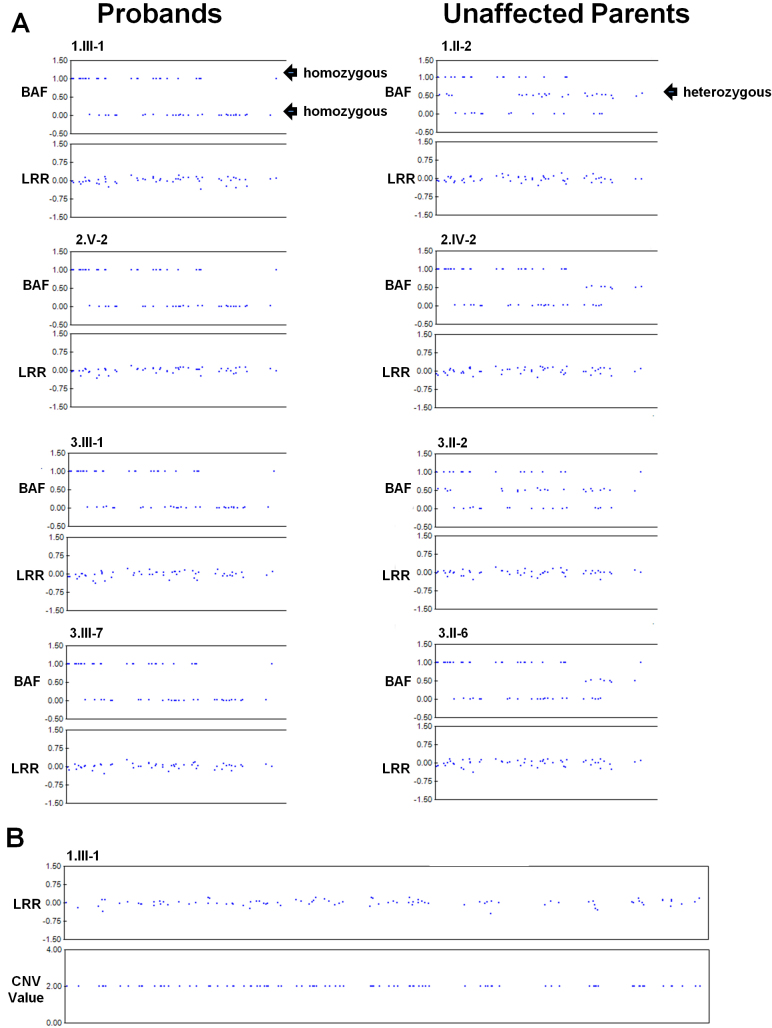
Montage illustrating representative genotype and copy number data across the IBD interval and *FREM1*. **A**: Genotype status (upper panels) and copy number data (lower panels) are provided for the first 55 SNPs in the IBD region (Chr9: 14,377,817–14,484,388). The BAF plots demonstrate homozygosity in the probands (BAF=1 or 0) and heterozygosity in the unaffected parents (BAF=0.5). The LRR plots also suggest no CNVs are present (LRR ~0). **B**: The lack of CNVs in *FREM1* (14,727,151–14,900,234) is evident from LRR plots. CnvPartition did not detect any CNVs in this region as all 96 SNPs in this region were assigned a normal CNV value of 2.

Only a small number of SNPs (67 – 95 [~0.01%]; Appendix 3) were excluded due to non-Mendelian errors, indicating that the genotyping data were of high quality. High homozygosity levels were observed in the four affected individuals (range: 9.3% – 15.8%; Appendix 4), indicating very substantial degrees of consanguinity that contrast with the ~6% theoretically calculated for the offspring of a first cousin marriage [34,35]. Homozygosity mapping analysis identified only a single segment that is identical by descent in the four affected individuals. This 330kb interval on 9p22.3 (Chr.9: 14,377,817 - 14,711,766, flanking SNPs rs2382470 and rs1494359) lies approximately 16 kb 3′ to the last exon of *FREM1* and the SNPs in this IBD interval display BAF values of 1 or 0 in the probands (demonstrating homozygosity) while the unaffected parents are heterozygous (BAF=~0.5) (Figure 3A; upper panels). Equally, the LRR values cluster around zero for the 152 SNPs in the IBD region, demonstrating the absence of any CNVs (Figure 3A; lower panels). In particular, the SNPs encompassing *FREM1* (9: 14,727,151 - 14,900,234) have normal LRR values and additional automated CNV analysis (CnvPartition) demonstrated that no CNVs were detectable in either the IBD (data not shown) or *FREM1* intervals (Figure 3B). Similarly, no CNVs or additional IBD regions were detected in the intervals encompassing *FRAS1*, *FREM2*, *FREM3*, or *GRIP1* (data not shown).

**Figure 4:**
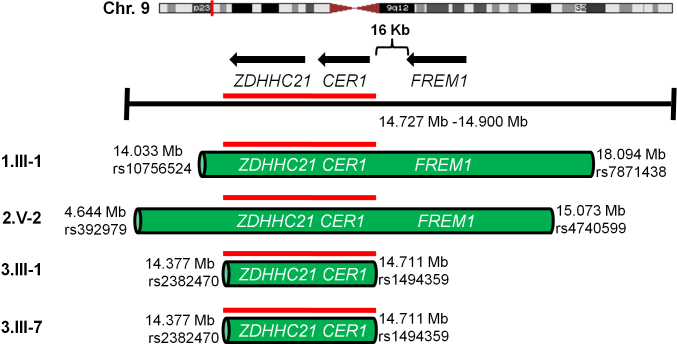
Illustration of the homozygous regions and the IBD interval in the four probands. The regions of homozygosity, which range from 330 kb to 10.4 Mb, include a 330 kb IBD interval common to all probands (red line). This interval contains *ZDHHC21* and *CER1*, and is 16 kb 3′ of *FREM1*’s last exon. Homozygosity mapping defined one IBD interval suggesting that mutation responsible for MOTA lies within the narrow 330 kb region.

The IBD region contains two genes *CER1* (a TGF-β signaling antagonist) [31] and *ZDHCC21* (a regulator of hair follicle development) [32] and as illustrated (Figure 4) its border is distinct from that of *FREM1*. Sequencing was performed initially for *CER1* and *ZDHCC21*, with no coding or splice site mutations identified. Notwithstanding the homozygosity mapping data, the 38 exons of *FREM1* were next sequenced and did not identify any causative variants. Ten homozygous variants were present: seven that result in synonymous amino acid substitutions, one non-synonymous SNP (A1212S) present in 28% of controls (dbSNP rs35870000), and a 5′UTR variant (Table 2). Notably a variant (c.5556A>G) that was recently described as contributing to MOTA [35], did not segregate in an autosomal recessive pattern (homozygous 1.III-1; heterozygous 3.III-1 and 3.III-7; homozygous wildtype 2.V-2). Seven non-coding regions within the IBD interval were found to be evolutionarily conserved with >70% identity between humans and *Xenopus*. Sequencing these seven regions identified a homozygous T>C base pair substitution that segregated with the disease phenotype (all probands: C/C, unaffected parents T/C; Appendix 5).

**Table 2:** Sequence variants identified.

**Gene**	**Exon**	**Variant**	**Amino acid residue**	**dbSNP reference number**
*ZDHHC21*	exon 6	c.318 T>C	C106C	rs17215796
*FREM1*	exon 3	c.-135C>G	N/A	
	exon 5	c.456 A>G	Q152Q	rs10961757
	exon 21	c.3634 G>T	A1212S	rs35870000
	exon 26	c.4785 C>T	A1595A	rs10733289
	exon 26	c.4791 T>C	D1597D	rs1032474
	exon 27	c.5004 C>A	I1668I	rs17219005
	exon 31	c.5556 A>G	G1853G	Not described
	exon 34	c.5859 T>C	V1953V	rs4741426

Note: no sequence variants were identified in *CER1* and the A1212S alteration in *FREM1* is present in 28% of controls (Coriell Collection).

## Discussion

This study’s key finding is the identification of a 330 kb region on chromosome 9p22.3 that is associated with MOTA syndrome. This illustrates the value of studying consanguineous populations such as the First Nations with homozygosity analysis. These findings localize the causative variant to an interval adjacent to *FREM1*, which represents an excellent candidate on the basis of the recapitulation of the human phenotypes in *Frem1* mutant mice [36,37], and related phenotypes induced by mutation of other *FRAS/FREM* gene family members. This study was predicated on the assumption that the level of homozygosity in a geographically isolated population, living on a remote reserve, would be increased. The range of autosomal homozygosity observed (9.3% - 15.8%), which in some cases exceeds that observed in other consanguineous populations or in the offspring of first cousin marriages [34], validates the approach used and contrasts with the far lower rates observed in a general population (1.9% - 4.6%) [38]. These data, derived from a very small number of affected individuals, illustrate the applicability of homozygosity mapping in the First Nations and suggest that it may permit other causes of this population’s disproportionately large disease burden to be identified.

While this manuscript was in preparation, two papers were published that substantially advanced understanding of *FREM1*’s role in these disorders [13,35]. The first, reported several *FREM1* mutations in MOTA cases of either First Nations or European ancestry [35] including: an inframe deletion of exons 8–23, one nonsense, two missense, and a synonymous alteration (c.5556A>G). Notably, neither of the two variants identified in First Nations patients (deletion of exons 8 – 23 or c.5556A>G [G1853G]) is the cause in our cases, in view of the absence of CNVs in the 330 kb region (Figure 3) and the fact that c.5556A>G’s does not segregate with disease (data not shown). Since a second causative allele was not identified in some individuals of Oji-Cree ancestry reported in Slavotinek et al. [35], the possibility therefore exists that a still to be identified allele is common to both the Oji-Cree and First Nations populations. The second publication describes heterozygous *FREM1* deletions and 3 missense variants that associate with metopic craniosynostosis as well as documenting the contribution of *FREM1* in patterning the murine cranial skeleton [13]. Accordingly our study demonstrates additional genetic heterogeneity among the First Nations, who would have been anticipated to have a single cause for the phenotype.

The most parsimonious explanation for our findings is that a sequence variant within the 330 kb IBD interval, which is located 16 kb 3′ of *FREM1*’s last known exon, causes MOTA. This is most likely to represent a regulatory element; however the possibility that an additional exon remains to be defined, cannot be excluded. Support for the concept of a regulatory variant is provided by the *Frem1^bfd^* murine strain, which lacks a coding *Frem1* mutation and is believed to have a variant in a control region that causes cryptophthalmos-like phenotypes [37]. *Frem1*’s role during development suggests that its temporal-spatial expression is tightly controlled, in keeping with the regulatory elements and tissue specific enhancers defined for a range of other developmental regulatory genes [39]. There are several examples of such mutated sequences in both ocular and systemic diseases, with regulatory mutations 3′ to *PAX6* causing aniridia and demonstrated to be functionally relevant by murine transgenesis rescue experiments [40,41]. In an attempt to define such elements, seven regions conserved across vertebrates were sequenced, identifying a homozygous T>C base pair substitution (ECR-7) that segregated with the phenotype. Bioinformatics analysis for regulatory elements using the VISTA Enhancer Browser online database [42] yielded no tissue specific enhancers for this region (data not shown). The most likely explanation is that this variant is in linkage disequilibrium with the true mutation, and it should be noted that sequence conservation is not necessarily a criteria of all regulatory elements [43]. Future research directions to support the relevancy of the identified 330 kb IBD region could include assaying *FREM1* expression from mRNA isolated from skin fibroblasts of MOTA probands, relative to a housekeeping gene and control samples. In parallel, next generation sequencing of the 330 kb IBD interval is increasingly feasible. It is interesting to note that the transcription factor delta-Np63 has been shown to control expression of members of the *Fras*/*Frem* gene family and displays enhancer activity in the murine nose, eyelids, genitals, and digits [44,45], the tissue domains affected in FS, BNAR, and MOTA.

In summary, this study extends *FREM1* heterogeneity in MOTA syndrome of First Nations ancestry. Homozygosity mapping defined one 330 kb IBD region on chromosome 9p22.3 comprising 152 SNPs in 4 probands. Sequencing the genes in or adjacent to this interval (*FREM1*, *CER1*, and *ZDHHC21*) revealed no disease-causing mutations. Accordingly, we infer that a variant within this region is responsible for MOTA syndrome, and suggest that future studies are indicated to define the causative mutation and by facilitating genetic counselling, reduce the high prevalence of MOTA syndrome in these isolated populations.

## Appendix 1. Primers used to amplify *CER1* and *ZDHHC21*.

To access the data, click or select the words “Appendix 1.” This will initiate the download of a compressed (pdf) archive that contains the file.

## Appendix 2. Primers used to amplify ECRs.

To access the data, click or select the words “Appendix 2.” This will initiate the download of a compressed (pdf) archive that contains the file.

## Appendix 3. Erroneous SNPs identified.

GenomeStudio’s heritability report algorithm was used to identify potentially discrepant parent-child relationships and reveal non-Mendelian genotyping errors. Erroneous SNPs were removed before homozygosity analysis with PLINK. More than 99.9% of SNPs are inherited in a Mendelian manner from unaffected mothers to probands verifying correct parent child relationship. To access the data, click or select the words “Appendix 3.” This will initiate the download of a compressed (pdf) archive that contains the file.

## Appendix 4. Percentage genome homozygosity.

Percentage homozygosity was derived from the ratio of the total length of all autosomal homozygous regions, divided by the length of all autosomes (2,867 Mb). To access the data, click or select the words “Appendix 4.” This will initiate the download of a compressed (pdf) archive that contains the file.

## Appendix 5. A homozygous point mutation within ECR-7 segregates with MOTA.

Electropherograms of a point mutation found to segregate with the disease are shown for the four probands and the unaffected parents in ECR-7, a region identified by ECR Browser to be conserved from human, chimpanzee, rhesus macaque, cow, opossum, rat, mouse, chicken and frog, but not in puffer fish or zebrafish. Probands are homozygous C/C while unaffected parents are C/T. The genomic reference used to compare sequence is T/T in this position. To access the data, click or select the words “Appendix 5.” This will initiate the download of a compressed (pdf) archive that contains the file.
